# The Impact of Nociception Monitor-Guided Multimodal General Anesthesia on Postoperative Outcomes in Patients Undergoing Laparoscopic Bowel Surgery: A Randomized Controlled Trial

**DOI:** 10.3390/jcm13020618

**Published:** 2024-01-22

**Authors:** Satoshi Okamoto, Hiroki Ogata, Shohei Ooba, Ayano Saeki, Fumiya Sato, Kazunori Miyamoto, Mayu Kobata, Hiroai Okutani, Ryusuke Ueki, Nobutaka Kariya, Munetaka Hirose

**Affiliations:** Department of Anesthesiology and Pain Medicine, Hyogo Medical University Faculty of Medicine, Nishinomiya, Hyogo 663-8501, Japan

**Keywords:** C-reactive protein, inflammation, nociception, nociceptive response, postoperative complications

## Abstract

Background: Excess surgical stress responses, caused by heightened nociception, can lead to elevated levels of postoperative inflammation, resulting in an increased incidence of complications after surgery. We hypothesized that utilizing nociception monitor-guided multimodal general anesthesia would exert effects on postoperative outcomes (e.g., serum concentrations of C-reactive protein (CRP) after surgery, postoperative complications). Methods: This single-center, double-blinded, randomized trial enrolled ASA class I/II adult patients with normal preoperative CRP levels, scheduled for laparoscopic bowel surgery. Patients were randomized to receive either standard care (control group) or nociception monitor-guided multimodal general anesthesia using the nociceptive response (NR) index (NR group), where NR index was kept below 0.85 as possible. The co-primary endpoint was serum concentrations of CRP after surgery or rates of 30-day postoperative complications (defined as Clavien–Dindo grades ≥ II). Main Results: One hundred and four patients (control group, *n* = 52; NR group, *n* = 52) were enrolled for analysis. The serum CRP level on postoperative day (POD) 1 was significantly lower in the NR group (2.70 mg·dL^−1^ [95% confidence interval (CI), 2.19–3.20]) than in the control group (3.66 mg·dL^−1^ [95% CI, 2.98–4.34], *p* = 0.024). The postoperative complication rate was also significantly lower in the NR group (11.5% [95% CI, 5.4–23.0]) than in the control group (38.5% [95% CI, 26.5–52.0], *p* = 0.002). Conclusions: Nociception monitor-guided multimodal general anesthesia, which suppressed intraoperative nociception, mitigated serum concentrations of CRP level, and decreased postoperative complications after laparoscopic bowel surgery.

## 1. Introduction

Intraoperative risks, in addition to inherent patient characteristics, contribute to postoperative outcomes [1]. As one of these intraoperative risks, surgical trauma stimulates the nociceptors of the somatosensory nervous system and immune cells, eliciting surgical stress responses in the form of neuroendocrine–metabolic and inflammatory–immune responses [2,3]. Although moderate surgical stress responses facilitate and support postoperative recovery, excessive surgical stress responses could potentially lead to higher levels of inflammation, increasing the incidence of postoperative complications [2,3].

The intraoperative management of general anesthesia by utilizing short-acting opioids and regional anesthesia while maintaining intraoperative normothermia, has been reported to counterbalance excessive surgical stress responses, contributing to the suppression of inflammatory responses [3,4]. Nociception, which is defined as the neural process of encoding noxious stimuli, induces autonomic (e.g., elevated blood pressure) or behavioral responses. A nociception monitor that reflects a balance between nociception caused by surgical trauma and anti-nociception provided by anesthesia may represent an aspect of surgical stress responses during surgery [5]. As a result, nociception monitor-guided general anesthesia would potentially contribute to the suppression of surgical stress responses, and may thus have the potential to suppress postoperative inflammation.

The nociceptive response (NR) index, as one of the nociception monitors used during general anesthesia, is a dimensionless number between 0 and 1, with higher values reflecting increased nociception. The NR index is calculated using three hemodynamic variables: heart rate (HR); systolic blood pressure (SBP); and perfusion index (PI) [6]. Observational studies have reported that a higher averaged NR index from the start to the end of surgery (mean NR) in patients undergoing gastrointestinal surgery under general anesthesia was associated with higher postoperative levels of serum C-reactive protein (CRP), a marker of inflammation [7]. Mean NR index < 0.85 during elective laparoscopic gastrointestinal surgery was reportedly associated with lower serum concentrations of CRP after surgery in addition to a reduced rate of postoperative complications [7]. Furthermore, mean NR index, along with preoperative CRP levels and duration of surgery, serves as a predictive factor for postoperative CRP levels in patients undergoing gastrointestinal surgery [5]. Thus, there might be a significant association between intraoperative nociception and postoperative CRP levels. However, there have been no reports of a randomized controlled trial investigating the effects of nociception monitor-guided general anesthesia on postoperative CRP levels and complications.

Our hypothesis was that nociception monitor-guided general anesthesia, utilizing multimodal strategies during surgery, would have an impact on either postoperative CRP levels or complications. This randomized controlled trial aims to test this hypothesis among patients undergoing elective laparoscopic bowel surgery. The purpose of the present study was to evaluate the effects of nociception monitor-guided general anesthesia on postoperative outcomes, but not the effects of different types of anesthetics and different methods of anesthesia on that.

## 2. Materials and Methods

### 2.1. Ethics

This prospective, double-blinded, parallel-arm, randomized controlled trial was approved by the Ethics Committee of Hyogo Medical University (Chairperson: Koichi Noguchi) on 21 January 2019 (approval no 3133). This study was registered before patient enrollment to the UMIN Clinical Trials Registry (UMIN000035415; principal investigator, Munetaka Hirose) on 1 March 2019, and was conducted at a single-site, tertiary teaching hospital in Japan to evaluate the effect of nociception monitor-guided multimodal general anesthesia on postoperative serum CRP levels. We amended patient conditions in our protocol from “abdominal malignant tumor” to “abdominal tumors and inflammatory diseases” on 29 August 2019 due to the unexpectedly slow recruitment speed.

### 2.2. Participants

Eligible patients were ≥20 years old of either sex and scheduled for elective laparoscopic small or large bowel surgery under general anesthesia for surgical treatment of either abdominal tumor or inflammatory bowel disease (IBD). Exclusion criteria were American Society of Anesthesiologists (ASA) class ≥ III or serum concentration of CRP before surgery ≥0.3 mg∙dL^−1^. Investigators evaluated the eligibility of each patient and obtained written informed consent for enrollment before surgery.

### 2.3. Randomization

Patients were randomized to receive nociception monitor-guided general anesthesia (NR group) or standard care (control group) using a computer-generated randomization list by a research assistant not involved in this study. Attending anesthesiologists were informed by the research assistant of the randomization assignment for the patient before the patient entered the operating room. Attending anesthesiologists were not blinded to patient group allocation, and therefore did not participate in either data acquisition after surgery or data analysis. The research assistant collecting data before and after surgery was blinded to group allocation. Patients, surgeons, and staff in the operation room and postoperative care unit were not informed of group allocations.

### 2.4. Perioperative Management for Standard Care

None of the patients received premedication. Standard monitoring was established, including electrocardiography, pulse oximetry, non-invasive blood pressure monitoring, and capnometry. General anesthesia was induced with propofol (1.5 mg∙kg^−1^), fentanyl (2 μg∙kg^−1^), and rocuronium (0.9 mg∙kg^−1^), followed by insertion of a tracheal tube, and was maintained with fentanyl, remifentanil, and rocuronium with sevoflurane or desflurane. Prophylactic antibiotics were administered intravenously within 60 min prior to surgical incision. An invasive blood pressure monitor was used by the attending anesthesiologists if appropriate. Doses of continuously infused remifentanil and bolus injection of fentanyl, in addition to injections of vasoactive agents such as ephedrine and phenylephrine, were adjusted to maintain SBP within a range of ±20% of the pre-anesthesia level as much as possible. Bispectral index (BIS) was maintained between 40 and 60 by adjusting the end expiratory concentration of sevoflurane or desflurane for sedation. Attending anesthesiologists administered additional bolus rocuronium as needed. Body core temperature, measured at the forehead using a thermometer (Bair Hugger Temperature Monitoring System; 3M, St. Paul, MN, USA), was maintained at 36.0–37.0 °C using a forced-air warming system. The methods for controlling mechanical ventilation while maintaining normocapnia were left to the discretion of the attending anesthesiologists.

Additional ultrasound-guided abdominal block (as transversus abdominis plane block and/or rectus sheath block) was performed after tracheal intubation if deemed appropriate by the attending anesthesiologists. Success of the block was determined by confirming adequate spread of the local anesthetic around the appropriate space with bolus injection of 0.25% levobupivacaine, using ultrasonography, and the nerve block needle was then pulled out. The total dose of levobupivacaine to be administered was confirmed to be below the maximum recommended dose before injection [8]. No catheter for continuous infusion was placed after these blocks.

During surgery, both intravenous infusion of dexamethasone for the prevention of postoperative nausea and vomiting and intravenous infusion of flurbiprofen, a nonsteroidal anti-inflammatory drug (NSAID) and/or acetaminophen for analgesia were also determined by attending anesthesiologists.

After surgery, all patients received continuous administration of intravenous fentanyl at 25–50 μg·h^−1^, along with oral administration of NSAIDs, acetaminophen, or tramadol, for postoperative analgesia for 2 days after surgery.

### 2.5. Nociception Monitor-Guided Anesthesia

In the NR group, NR index was utilized for nociception monitoring in the present study. NR index, which is calculated using the three variables of heart rate (HR), systolic blood pressure (SBP), and perfusion index (PI), represents intraoperative nociception using a dimensionless number between 0 and 1 [9]. The NR index has been developed using a differential equation model for describing logistic function assessing associations between the intraoperative stimulation (S) and its physiological responses (R). The differential equation is

$$\frac{dR}{dS}=kR(1-\frac{R}{R_{max}})$$

where R_max_ is the maximum response, 1 − R/R_max_ represents the suppression of R by feedback regulations from the descending pain inhibitory system and baroreflex, and k is a constant. To solve this equation, the S value was determined to be S = 0.01HR + 0.02SBP − 0.17PI by analyzing three hemodynamic variables, namely HR, SBP, and PI at the time of skin incision, to most effectively discriminate the three nociception levels between tympanoplasty, laparoscopic cholecystectomy, and gastrectomy [9]. After solving this differential equation, the NR formula was obtained as the following logistic function:

$$NR=\frac{2}{1+e^{-(0.01HR+0.02SBP-0.17PI)}}-1$$

the values of which indicate surgical stress responses according to intraoperative nociception with feedback regulations (Japanese Patent No. 6934251) [9].

NR index was calculated every 1 min during surgery. PI values were derived from a plethysmographic pulse wave amplitude via pulse oximetry. SBP values were obtained from non-invasive or invasive blood pressure monitoring. When SBP was measured every 5 min during non-invasive measurement, the latest SBP value was used to calculate NR index every 1 min. The equation for NR index was installed on the anesthesia information managing system (ORSYS; Philips Japan, Tokyo, Japan). NR index was shown every 1 min on the anesthesia record screen in the NR group, and is recorded every 1 min in our institutional anesthesia database (ORSYS; PHILIPS, Tokyo, Japan).

In order to compare the effects of nociceptive response-guided anesthesia between the control and NR groups, the method of multimodal anesthesia was not fixed and was left to the anesthesiologist in charge in both groups. After patient randomization, attending anesthesiologists in the NR group were instructed by the investigator to control NR index to <0.85 as much as possible with multimodal strategies [10], including short-acting opioids, NSAIDs, regional anesthesia, and vasoactive agents [5]. The dose of either fentanyl or remifentanil was thus increased in the event of transient increases in NR index to ≥0.85 during surgery. Landiolol, an ultra-short-acting β1-selective antagonist, was also administered, if both anesthetics and analgesics proved insufficient to suppress NR index during surgery. SBP was maintained within the range of ±20% of the pre-anesthesia level as much as possible.

In the control group receiving standard care, however, the anesthesia record screen did not display NR index, and the attending anesthesiologists did not refer to it during anesthesia, even though NR index was calculated and recorded every 1 min in our institutional anesthesia database.

### 2.6. Outcome Measurements

Co-primary endpoint of this study was the serum concentration of CRP after surgery or the rate of 30-day postoperative complications. Secondary endpoints were changes in NR index during surgery and acute postoperative pain as assessed while the patient was at rest.

### 2.7. Demographic Characteristics and Preoperative Risk Assessments for Postoperative Complications

Demographic information was collected after enrollment by the research assistant. Serum concentration of CRP was measured using a latex turbidimetric immunoassay (reference range, 0.00–0.30 mg·dL^−1^). Preoperative risks for postoperative outcomes were assessed, using preoperative prediction models comprising the Surgical Outcome Risk Tool (SORT) and American College of Surgeons National Surgical Quality Improvement Program (ACS NSQIP) [11,12]. Predicted risk in the SORT was calculated using variables of ASA, urgency, severity of surgery, malignancy, and age [11]. The ACS NSQIP Surgical Risk Calculator uses preoperative data from participant demographics (surgical procedure, age, sex, body mass index, functional status, emergency, ASA, steroid use, and current smoker status) and comorbid conditions (ascites, systemic sepsis, ventilator dependency, disseminated cancer, diabetes, hypertension requiring medication, congestive heart failure, dyspnea, severe chronic obstructive pulmonary disease, dialysis, and acute renal failure) [12].

### 2.8. Intraoperative Variables

Hemodynamic (e.g., SBP, HR, PI) values in addition to NR index were also recorded every 1 min in our institutional anesthesia database (ORSYS; PHILIPS, Tokyo, Japan). Blood pressure values, however, were recorded every 5 min in the case of non-invasive blood pressure measurement and every 1 min in the case of invasive blood pressure measurement. Highest, lowest, and mean values of these physiological variables during surgery in each patient were also obtained using Vi-Pros data search software (Dowell, Sapporo, Japan). Mean blood pressure (MBP) below 65 mmHg and below 55 mmHg during surgery were also recorded, as these thresholds have been linked to increased risk of postoperative complications [13,14,15].

### 2.9. Postoperative Outcomes

Postoperative inflammatory responses and postoperative complications after surgery were assessed. Postoperative inflammatory status was assessed using serum CRP levels on postoperative day (POD) 1, which were identified as postoperative risk factors for postoperative outcomes on POD 1 [16,17]. The intensity of postoperative pain at surgical sites was evaluated at rest using an 11-point (scores 0–10) numerical rating scale (NRS) on POD 1.

The severity of postoperative complications was graded using the Clavien-Dindo classification [18]. This classification includes seven grades: grade I, any deviation from the normal postoperative course (allowed therapeutic regimens are drugs as antiemetics, antipyretics, analgesics, diuretics, electrolytes, and physiotherapy); grade II, alteration of the normal postoperative course (requiring pharmacological treatment with drugs other than such allowed for grade I complications); grade III, complications that require interventions under local anesthesia (IIIa) or general/epidural anesthesia (IIIb); grade IV, life-threatening complications with single-organ (IVa) or multiorgan dysfunction (IVb); and grade V, death within 30 days after surgery. Postoperative complications were defined as Clavien–Dindo grade ≥ II in the present study.

### 2.10. Sample Size Calculation

Sample size was calculated using PS Power and Sample Size Calculations version 3.0 software (Dupont WD and Plummer WD, Vanderbilt University Medical Center, Nashville, TN, USA). Calculations were performed assuming a type I error probability of 0.05 and power of 0.8. A previous observational study reported that serum concentrations of CRP on POD1 were 2.56 ± 2.34 mg·dL^−1^ in the low NR group (mean NR < 0.85) and 3.91 ± 2.37 mg·dL^−1^ in the high NR group (mean NR ≥ 0.85) undergoing elective laparoscopic gastrointestinal surgery [7]. Under the assumption that postoperative CRP levels would be 2.6 mg·dL^−1^ and 3.9 mg·dL^−1^ in patients from the NR and control groups, sample size was estimated to be 50 participants in each group (*n* = 100 in total). On the other hand, another previous study reported that the incidence of postoperative complications, defined as Clavien–Dindo grade ≥ II, was 26.2% in patients undergoing laparoscopic colorectal surgery [19]. Under the assumption that the probabilities of postoperative complications were 12% and 36% in patients from the NR and control groups, respectively, required sample size was estimated to be 49 participants in each group (*n* = 98 in total). We thus selected a total sample size of 100 to assess postoperative CRP levels or 30-day complications. To account for a potential 5% dropout rate, we planned to recruit at least 105 participants for this study.

### 2.11. Statistical Analysis

Data are presented mean ± SD, median and interquartile range, or frequency with percentage. Standardized mean differences were reported for comparisons of demographic characteristics. Each variable was evaluated using the unpaired t-test, the Mann–Whitney U test, or χ^2^ test as appropriate, and *p* < 0.05 was considered significant. On the other hand, values of co-primary endpoint with *p* < 0.025 were considered statistically significant after adjusting for multiple comparisons using the Bonferroni correction (*p* < 0.05/2). To compare differences in NR index between groups, NR index was analyzed at each point using an unpaired t-test with Bonferroni adjustment, where values of *p* < 0.008 were considered significant after correcting for multiple comparisons, given that seven parameters were tested (0.05/6 ≈ 0.008). Data analysis was performed using JMS Pro version 14.2.0 (SAS Institute Inc., Cary, NC, USA).

## 3. Results

**Figure 1:**
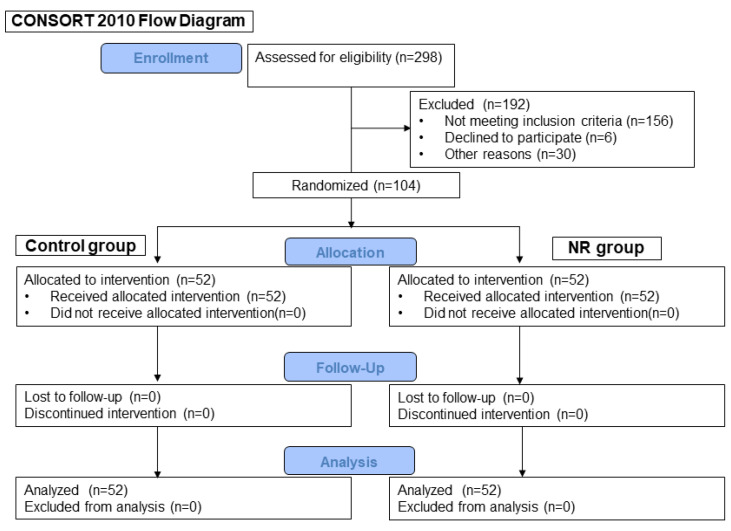
Consolidated Standards of Reporting Trials (CONSORT) flow diagram.

From March 2019 to February 2023, a total of 298 patients were screened for eligibility. Of these, 192 patients were excluded due to inclusion criteria, declining to participate, or unavailability of staff. As a result, 104 patients were enrolled and randomized to the control (*n* = 52) and NR groups (*n* = 52) (Figure 1). After we confirmed that no data were missing for primary or secondary endpoints, and no harmful events occurred during the study, we stopped the patients’ enrollment.

### 3.1. Preoperative Variables

Table 1 presents the demographic characteristics of participants showing no significant differences between groups. In preoperative risk assessments for postoperative outcomes, predicted risk of postoperative mortality and morbidity calculated using the SORT was almost identical between groups. Predicted risks of any or serious complications calculated using the ACS NSQIP Surgical Risk Calculator, however, were slightly higher in the NR group than in the control group.

### 3.2. Intraoperative Variables

No surgeries were converted to open surgery. The NR group received a significantly higher continuous dose of remifentanil and showed higher uses of flurbiprofen and landiolol compared to the control group (Table 2).

**Figure 2:**
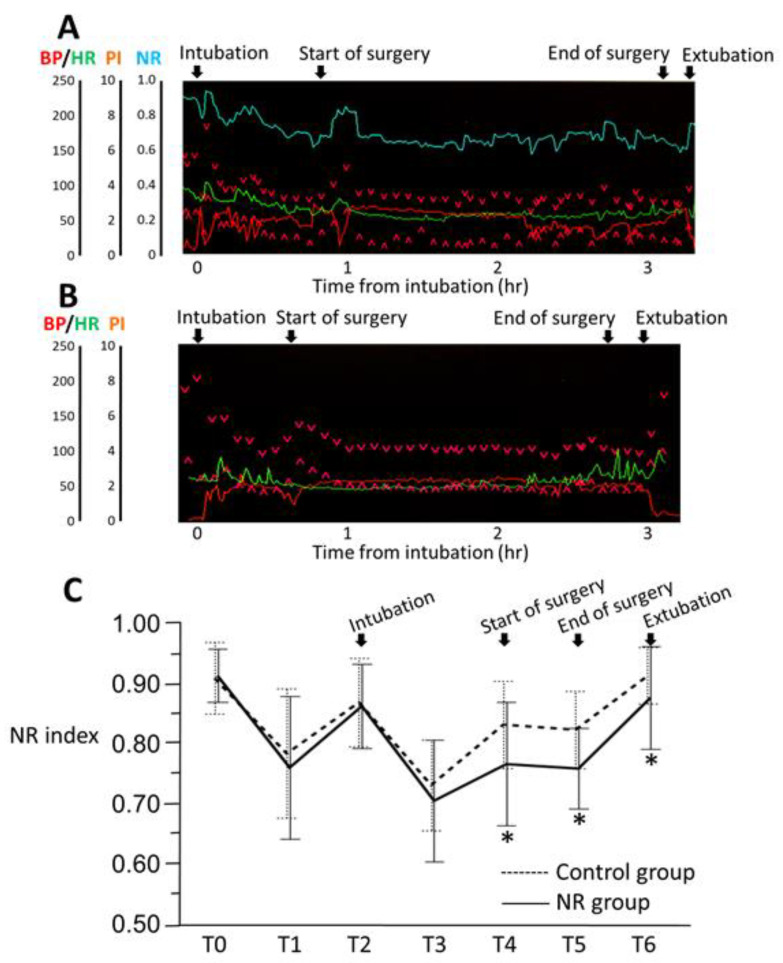
Typical anesthetic records and changes in NR index. Typical anesthetic records in the NR (**A**) and control (**B**) groups. Changes in NR index before induction of anesthesia (T0), at loss of consciousness (T1), at tracheal intubation (T2), before start of surgery (T3), at start of surgery (T4), at end of surgery (T5), and at eye opening (T6), in the NR (solid line) and control (dotted line) groups (**C**). Error bars indicate the standard deviation. * *p* < 0.008, significant difference using unpaired *t*-test with Bonferroni adjustment. BP, blood pressure; HR, heart rate; NR, nociceptive response; PI, perfusion index.

Typical anesthesia record screens in the NR and control groups are shown in Figure 2A and Figure 2B, respectively. The NR group showed significant suppression of both highest and mean NR values during surgery compared to the control group. However, lowest NR did not differ significantly between groups (Table 2). Figure 2C shows changes in NR index from the time before induction of anesthesia (T0) to eye opening after surgery (T6). Although no significant differences in NR index were evident between groups at T0 (*p* = 0.680), loss of consciousness (T1; *p* = 0.293), tracheal intubation (T2; *p* = 0.641), or before the start of surgery (T3; *p* = 0.139), NR index was significantly lower in the NR group when surgery was started (T4; *p* < 0.001), at the end of surgery (T5; *p* < 0.001), and at eye opening (T6; *p* = 0.008) compared to those in the control group.

On the other hand, here were no significant differences in the number of patients both with the lowest MBP below 55 mmHg and with that below 65 mmHg between groups (Table 2).

### 3.3. Postoperative Outcomes

A significant difference was apparent between NR and control groups with lower CRP levels on POD 1 (*p* = 0.024) (Table 3). Table 3 also displays the incidence of postoperative complications in each group. The incidence of postoperative complications (Clavien–Dindo grades ≥ II) was significantly lower in the NR group (11.5% [95% confidence interval (CI), 5.4–23.0]) than in the control group (38.5% [95% CI, 26.5–52.0]) (relative risk, 0.30 [95% CI, 0.13–0.69]; *p* = 0.002). Although the rates of postoperative complications classified as Clavien–Dindo grade II were comparable between the two groups (*p* = 0.047), those classified as Clavien–Dindo grade III was significantly lower in the NR group than in the Control group (*p* = 0.012). No postoperative complications classified as Clavien–Dindo grade IV or V were observed (Table 3).

Postoperative complications classified as Clavien–Dindo grade II in the control group (*n* = 14) were bleeding (*n* = 2), delirium (*n* = 2), liver dysfunction (*n* = 3), persistent fever (*n* = 2), pneumonia (*n* = 2), surgical site infection (*n* = 2), and others (*n* = 1). In contrast, those in the NR group (*n* = 6) included liver dysfunction (*n* = 1), persistent fever (*n* = 1), surgical site infection (*n* = 1), and others (*n* = 3). Although postoperative complications with a Clavien–Dindo grade of III in the Control group (*n* = 6) included ileus (*n* = 4) and anastomotic leakage (*n* = 2), there were no postoperative complications with a Clavien–Dindo grade of III in the NR group. However, there was no significant difference in the incidence of any complication between the two groups.

Acute postoperative pain at rest on POD 1, as assessed by NRS, was also significantly suppressed in the NR group compared to the control group (*p* < 0.001) (Table 3). Duration of hospitalization, however, showed no significant difference between NR and control groups (median (interquartile rang) = 10 (9–15) days and 13 (9–19) days, respectively, *p* = 0.058).

## 4. Discussion

The present study found that the NR group achieved significantly lower levels of postoperative CRP levels with a lower incidence of postoperative complications compared to the control group, despite similar preoperative predicted risks for postoperative complications between groups.

The postoperative CRP level on POD 1 was 3.66 mg·dL^−1^ in the control group, which was significantly higher than the level of 2.70 mg·dL^−1^ in the NR group. Lower mean NR is also reportedly associated with lower serum CRP levels on POD 1 after gastrointestinal surgery in our previous observational studies [7]. In a prior study involving adult patients undergoing bariatric surgery, the postoperative CRP level on POD 1 also showed a significant difference between 4.8 mg·dL^−1^ and 2.9 mg·dL^−1^ in patients with and without 30-day postoperative complications, respectively [20]. Additionally, there was a significant difference in the postoperative CRP level on POD 1 between 13.7 mg·dL^−1^ and 9.6 mg·dL^−1^ in adult patients undergoing elective minimally invasive colorectal surgery with and without postoperative complications (Clavien–Dindo grades ≥ III), as reported in a different study [16]. Another previous study also has shown that lower CRP levels on POD 1 were associated with lower incidences of postoperative complications after laparoscopic surgery [17]. Although preoperative morbidity and surgery-related factors have strong evidence for a link with postoperative complications, whereas anesthesia-related factors have been considered to show only moderate to weak evidence [21], an inhibitory effect of anesthesia on both postoperative inflammatory responses and complications may have been revealed by the equalization of other perioperative factors between groups in the present study. Since excessive inflammatory responses inducing edema, ischemia, and infection, impair wound healing and promote postoperative complications [22], the suppression of postoperative increases in CRP levels in the present study suggests mechanisms for the suppressive effects of nociception monitor-guided multimodal general anesthesia on postoperative complications. Thus, the significant decreases in postoperative CRP levels observed in the NR group compared to the control group in the present study thus strengthen the notion that suppression of acute inflammatory responses within a few days after surgery under nociception monitor-guided multimodal general anesthesia reduced 30-day postoperative complications after laparoscopic bowel surgery [5,16,17].

Previous observational studies have suggested that regional anesthesia, fentanyl, and remifentanil would be effective to suppress the NR index during surgery under general anesthesia [5]. In the present study, although significant differences in uses of remifentanil, flurbiprofen, and landiolol were seen, no significant difference in uses of fentanyl and regional anesthesia were identified between the control and NR groups. Multimodal general anesthesia suppressing the NR index to <0.85 by integrating these effects might suppress nociception and inflammation, which might reduce excessive surgical stress responses and decrease postoperative complications.

The mechanisms by which nociception monitor-guided multimodal general anesthesia suppressed serum CRP levels after surgery remain unclear. Short-acting opioids and regional anesthesia have anti-nociceptive effects, and NSAIDs have anti-inflammatory effects. In addition, β-antagonists exert both anti-inflammatory and anti-nociceptive effects [23]. Acute-phase responses to surgical trauma causes the expression of CRP by the liver, stimulated by pro-inflammatory cytokines (interleukin-6 and interleukin-1β) released from inflammatory and immune cells. Monocytes, in addition to platelets and endothelial cells, are activated by inflammation and induced to perform pro-inflammatory functions by CRP. The pro-inflammatory properties of CRP then activate neutrophils, monocytes, endothelial cells, and platelets, producing pro-inflammatory cytokines and thereby facilitating further production of CRP [24]. Pro-inflammatory cytokines also induce hematopoiesis by directly activating bone marrow [25]. Meanwhile, bone marrow is innervated by sympathetic and nociceptive nerves [26,27]. Increases in sympathetic activity stimulate α1-, α2-, and β2-receptors at sympathetic nerve terminals and increase norepinephrine levels, which contribute to hematopoiesis and an increase in white blood cell counts with an elevated proportion of neutrophils and monocytes [27,28], and cause tissue damage [29]. In the context of nociception monitor-guided multimodal general anesthesia, the use of short-acting opioids may potentially inhibit acute-phase responses in CRP levels through their anti-nociceptive effects. Additionally, we assume that the administration of NSAIDs might also reduce acute-phase responses by virtue of their anti-inflammatory properties. The latter subject warrants further investigation in future studies.

The effects of nociception monitor-guided anesthesia on postoperative outcomes have been investigated. The surgical pleth index (SPI), calculated using heartbeat interval and photoplethysmographic waveform amplitude, serves as a nociception monitor under general anesthesia [30]. SPI-guided general anesthesia has been reported to reduce the incidence of postoperative delirium after emergency noncardiac surgery [31] and laparoscopic colorectal surgery [32]. However, in the present study, there was no significant difference in the incidence of postoperative delirium between the NR and control groups.

Nociception monitor-guided multimodal general anesthesia also suppressed acute postoperative pain in the present study. Although the precise mechanisms of postoperative analgesia are unclear, postoperative anti-inflammatory states including lower CRP levels may be related. Further investigation is needed to precisely evaluate the effects of nociception monitor-guided multimodal general anesthesia on acute and chronic postsurgical pain.

One limitation of this study was that the investigation was restricted to patients without serious preoperative comorbidity undergoing only laparoscopic bowel surgery. Postoperative complications were more severe in patients with a higher mean NR index during thoracic surgery, where this mean NR index, reflecting the degree of surgical stress response, was considerably higher than those observed in laparoscopic gastrointestinal surgery [5]. During surgeries with very high levels of surgical stress, however, the impact of anesthesia-related factors on postoperative complications may be masked by factors related to the surgery itself. Whether nociception monitor-guided multimodal anesthesia has inhibitory effects on postoperative inflammatory responses and complications after surgeries with very high levels of surgical stress remains unclear. The second limitation of this study was that the associations between short-acting opioid doses and postoperative complications are not yet understood. The present study suggested that sufficient doses of opioids to suppress nociception during surgery would be beneficial for the prevention of postoperative complications. While no evidence suggests that opioid-sparing or opioid-free strategies for multimodal general anesthesia are beneficial in terms of preventing postoperative complications [33], further investigation is needed to clarify the precise mechanisms by which high intraoperative doses of remifentanil, suppressing the NR index, prevented postoperative inflammatory responses and complications in the present study. The third limitation of the present study was that it included patients with either abdominal tumors or IBD without information of history of abdominal surgery, who had normal preoperative serum CRP levels (<0.3 mg∙dL^−^^1^). Preoperative CRP levels have been shown to influence postoperative complications after gastrointestinal surgery [5], and preoperative CRP levels ≥1.45 mg∙dL^−1^ are reportedly associated with severe postoperative complications in IBD patients [34]. By focusing on patients with normal CRP levels before surgery, our study successfully identified the effects of nociception monitor-guided anesthesia on postoperative CRP levels. However, future research should investigate these effects in patients with higher preoperative CRP levels with information of history of abdominal surgery. The fourth limitation of this study was that the NR group received a higher use of flurbiprofen, an NSAID, compared to the control group. This disparity in NSAID usage may have influenced postoperative CRP levels in the NR group. Therefore, future investigations should focus on patients receive equivalent uses of NSAID in both groups. The last limitation was that the NR index cannot be utilized in awake patients, since it increases higher in the order of higher levels of consciousness [35]. Thus, the results of the present study cannot be applied to awake patients under regional anesthesia.

## 5. Conclusions

ASA I/II patients with normal preoperative CRP levels, undergoing elective laparoscopic bowel surgery with nociception monitor-guided multimodal general anesthesia, had lower inflammatory responses and a reduced incidence of postoperative complications compared to the control group. Postoperative reductions in serum CRP levels after surgery via mitigation of intraoperative nociception likely contributed as mechanisms suppressing postoperative complications.

## Figures and Tables

**Table 1:** Demographic characteristics of participants and preoperative risk assessments for postoperative complications.

Preoperative Variables	Control Group (*n* = 52)	NR Group (*n* = 52)	Standardized Mean Difference
Demographic characteristics			
Age, yr	57 ± 19	56 ± 19	0.09
Male sex, *n*	33 (63.5%)	31 (59.6%)	0.08
BMI, kg·m^−2^	21.8 ± 3.6	21.6 ± 3.0	0.06
ASA classification			
I, *n*	10 (19.2%)	9 (17.3%)	0.05
II, *n*	42 (80.8%)	43 (82.7%)	0.05
Underlying pathology			
Abdominal tumor, *n*	29 (55.8%)	35 (67.3%)	0.08
IBD, *n*	23 (44.2%)	17 (33.7%)	0.08
CRP level, mg·dL^−1^	0.07 (0.03–0.14)	0.06 (0.02–0.14)	0.06
Preoperative risk assessments			
ACS NSQIP Surgical Risk Calculator Prediction of any complication, % Prediction of serious complication, %	6.9 (6.1–8.0) 5.9 (4.9–6.8)	7.1 (6.5–7.9) 5.9 (5.4–6.8)	0.27 0.23
SORT			
Predicted risk	0.0037 (0.0019–0.0079)	0.0037 (0.0019–0.0079)	0.11

Values are represented as means ± SD, median (interquartile range), or *n* (%). A standardized mean difference >0.1 was considered indicative of a significant difference. ACS NSQIP, American College of Surgeons National Surgical Quality Improvement Program; ASA, American Society of Anesthesiologists; BMI, body mass index; CRP, C-reactive protein; IBD, inflammatory bowel disease; NR, nociceptive response; SORT, Surgical Outcome Risk Tool.

**Table 2:** Intraoperative variables.

Characteristic	Control Group (*n* = 52)	NR Group (*n* = 52)	*p* Value
Surgical and Anesthetic Data			
Laparoscopic surgery			
Small/large bowel resection, *n*	4/48 (7.7%/92.3%)	7/45 (13.5%/86.5%)	0.339
Invasive blood pressure monitor, *n*	13 (25.0%)	11 (21.2%)	0.642
Regional anesthesia, *n*	33 (63.5%)	41 (78.9%)	0.083
Anesthetics and analgesics used			
Continuous doses of remifentanil, μg·kg^−1^·min^−1^ Total doses of fentanyl, μg·kg^−1^·min^−1^ Total doses of rocuronium, mg·kg^−1^·min^−1^ Dexamethasone, *n* Flurbiprofen, *n* Acetaminophen, *n*	0.169 ± 0.045 0.021 ± 0.008 6.31 ± 3.13 46 (88.5%) 26 (50.0%) 46 (88.5%)	0.196 ± 0.062 0.022 ± 0.008 6.47 ± 2.66 49 (94.2%) 37 (71.2%) 44 (84.6%)	0.011 * 0.596 0.770 0.295 0.027 * 0.566
Vasoactive drugs used			
Phenylephrine, *n*	28 (53.9%)	26 (50.0%)	0.695
Ephedrine, *n*	34 (65.4%)	32 (61.5%)	0.684
Landiolol, *n*	0 (0.0%)	5 (9.6%)	0.022 *
Duration of surgery, min	241 ± 98	218 ± 107	0.292
Total fluid input, mL	1625 (1300–2188)	1500 (1300–1838)	0.373
Blood loss, mL	25 (0–68)	10 (0–30)	0.128
Urine volume, mL	210 (100–300)	180 (100–350)	0.293
Mean BIS	45 ± 6	43 ± 7	0.207
Mean core temperature, °C	36.7 ± 0.4	36.7 ± 0.4	0.963
NR			
Highest NR	0.919 ± 0.041	0.875 ± 0.059	<0.001 **
Mean NR	0.809 ± 0.047	0.753± 0.060	<0.001 **
Lowest NR	0.649 ± 0.134	0.616 ± 0.090	0.146
Lowest MBP			
Lowest MBP < 55 mmHg	14 (26.9%)	15 (28.9%)	0.827
Lowest MBP < 65 mmHg	34 (65.4%)	42 (80.8%)	0.077

Values are represented as means ± SD, median (interquartile range), or *n* (%). Significant differences at * *p* < 0.05. or ** *p* < 0.01. BIS, bispectral index; HR, heart rate; MBP, mean blood pressure; NR, nociceptive response.

**Table 3:** Postoperative outcomes.

Outcomes	Control Group (*n* = 52)	NR Group (*n* = 52)	*p* Value
Postoperative CRP levels on POD			
CRP level, mg·dL^−1^	3.66 [95% CI, 2.98–4.34]	2.70 [95% CI, 2.19–3.20]	0.024 *
Postoperative complications within 30 days after surgery			
Clavien–Dindo grade ≥ II	20 (38.5% [95% CI, 26.5–52.0])	6 (11.5% [95% CI, 5.4–23.0])	0.002 **
Clavien–Dindo grade II	14 (26.9% [95% CI, 16.8–40.3])	6 (11.5% [95% CI, 5.4–23.0])	0.047
Clavien–Dindo grade III	6 (11.5% [95% CI, 5.4–23.0])	0 (0.0%)	0.012 *
Postoperative pain on POD			
NRS at rest	4 (3–5)	1 (1–2)	<0.001 **

Values are represented as mean [95% CI], median (interquartile range), or *n* (%). Significant differences at * *p* < 0.025 or ** *p* < 0.005. CI: confidence interval, CRP: C-reactive protein, NR: nociceptive response, NRS, numerical rating scale; POD: postoperative day.

## Data Availability

The data that support the findings of this study are available on request from the corresponding author.
